# Risk-Adjusted Analysis of Relevant Outcome Drivers for Patients after More Than Two Kidney Transplants

**DOI:** 10.1155/2015/712049

**Published:** 2015-02-01

**Authors:** Lampros Kousoulas, Florian W. R. Vondran, Paulina Syryca, Juergen Klempnauer, Harald Schrem, Frank Lehner

**Affiliations:** General, Visceral and Transplant Surgery, Hanover Medical School, 30625 Hanover, Germany

## Abstract

Renal transplantation is the treatment of choice for patients suffering end-stage renal disease, but as the long-term renal allograft survival is limited, most transplant recipients will face graft loss and will be considered for a retransplantation. The goal of this study was to evaluate the patient and graft survival of the 61 renal transplant recipients after second or subsequent renal transplantation, transplanted in our institution between 1990 and 2010, and to identify risk factors related to inferior outcomes. Actuarial patient survival was 98.3%, 94.8%, and 88.2% after one, three, and five years, respectively. Actuarial graft survival was 86.8%, 80%, and 78.1% after one, three, and five years, respectively. Risk-adjusted analysis revealed that only age at the time of last transplantation had a significant influence on patient survival, whereas graft survival was influenced by multiple immunological and surgical factors, such as the number of HLA mismatches, the type of immunosuppression, the number of surgical complications, need of reoperation, primary graft nonfunction, and acute rejection episodes. In conclusion, third and subsequent renal transplantation constitute a valid therapeutic option, but inferior outcomes should be expected among elderly patients, hyperimmunized recipients, and recipients with multiple operations at the site of last renal transplantation.

## 1. Introduction

Renal transplantation is the treatment of choice for patients with end-stage renal disease, as it increases the survival of the recipients but also improves their quality of life as compared to long-term dialysis treatment [1–5]. Despite great advances in the field of renal transplantation, transplant immunology, and immunosuppression, long-term renal allograft survival is still limited with an estimated half-life of about 9 years for primary deceased donor kidney grafts [6–8]. Therefore, most transplant recipients will face graft loss and return to dialysis treatment and many of them will be considered for a kidney retransplantation. Renal transplant recipients undergoing retransplantation display improved survival compared with those undergoing dialysis after graft failure [9–11], but the operative procedure still represents a surgical challenge with technical difficulties, especially in the case of a third or a fourth renal transplantation, as the new renal graft has to be positioned in previously manipulated fossae iliacae [12–15]. Moreover, recipients of multiple renal grafts constitute a unique population with high risk of complications and graft loss due to hyperimmunization and multiple comorbidities such as severe atherosclerosis with calcifications of the aortoiliac vessels [16, 17]. These factors are associated with poor patient and graft survival after multiple renal transplantations [18–20]. Given the shortage of donor kidneys and the increasing number of patients on the waiting list for renal transplantation, it is important to assess the patient and graft survival after second or subsequent renal transplantation and identify the factors that lead to inferior outcomes compared to those after primary and secondary kidney transplantation.

## 2. Patients and Methods

### 2.1. Setting and Type of the Study

This is a single-center, retrospective, observational study from a German kidney transplant center within the Eurotransplant community with institutional experience with more than 6000 kidney transplants since 1968.

### 2.2. Inclusion Criteria

Included into the study were all consecutive adult patients who received more than two kidney transplants in our institution between January 1990 and December 2010.

### 2.3. Exclusion Criteria

No exclusion criteria were defined for this study.

### 2.4. Study End Points

Primary study end points were defined as patient and graft survival after the last of multiple renal transplants with graft survival censored for death with functioning graft.

### 2.5. Patient Characteristics

The mean age of all included patients (*n* = 61) was 39 years (range 20–63 years). 32 patients were male and 29 female (52% versus 48%, resp.). 15 patients had blood group 0, 35 blood group A, two blood group B, and nine blood group AB. 59 patients were kidney-transplanted three times, nine patients four times, and three patients five times during follow-up. Their mean time on dialysis was 132.89 months (range 40–315 months). Regarding the cardiovascular risk profile of the recipients, it has to be mentioned that 52 patients (85%) were treated for hypertension, 20 patientes (33%) for diabetes and two patients (3%) had a history of myocardial infarction. Long-term follow-up in this series was between 0.5 and 22.8 years (mean: 10.0 years; median: 8.3 years). Patient characteristics are shown in Table 1.

### 2.6. Statistical Methods

This is an analysis of prospectively stored and retrospectively compiled data. Age at transplant, recipient blood group, recipient sex, maximum number of kidney transplants, maximum preformed antibodies in percent, maximum preformed antibodies in percent divided into groups (0–30%, >30%–70%, and >70%), preformed antibodies at the time of transplant in percent, preformed antibodies at the time of transplant in percent divided into groups (0–30%, >30%–70%, and >70%), number of HLA-DR mismatches, number of all HLA mismatches (HLA-A, -B, and -DR loci), number of HLA mismatches dived into groups (0–2, 3–6), perioperative plasmapheresis (yes/no), induction therapy (yes/no), type of induction therapy, cyclosporine versus tacrolimus based initial immunosuppression, mycophenolate mofetil versus azathioprine treatment, living donor versus deceased donor transplant, simultaneous nephrectomy of previous renal allograft (yes/no), number of previous operations at the site of the last renal transplantation, operating time in minutes, cold ischemic time in minutes, posttransplant surgical complications (yes/no), number of posttransplant surgical complications, reoperation due to a complication (yes/no), primary graft nonfunction (yes/no), acute rejection episodes after transplant (yes/no), chronic allograft rejection after transplant (yes/no), and graft loss censored for death with functioning graft (yes/no) were analyzed as possible risk factors with influence on primary and secondary study end points. All of these variables refer to the last of multiple kidney transplants in all patients. Their relevance was identified with univariate binary regression and Cox regression analysis, respectively. Variables with an alpha-level <0.05 were considered for risk-adjusted multivariate Cox regression and multivariate binary logistic regression analysis, which were performed to examine variations in risk according to patient and graft survival, acute rejection after transplantation, and primary graft nonfunction after transplantation, respectively. Kaplan-Meier analysis with log rank tests, Pearson's *χ* ^2^ test, and ANOVA pairwise Kruskal-Wallis tests were applied where appropriate. Additional evaluation of identified prognostic variables was subjected to ROC-curve analysis with determination of the area under the ROC-curve (AUROC) for the prediction of primary study end points where appropriate. Cut-off values for continuous variables were derived with the best Youden index based on ROC-curve analysis results where appropriate. For all statistical tests a *P* value <0.05 was defined as significant. The IBM SPSS statistics-software version 21.0 was used to perform statistical analysis.

## 3. Results

### 3.1. Patient Survival

Actuarial patient survival was 98.3% after one year, 94.8% after three years, and 88.2% after 5 years, respectively. Univariate Cox regression showed that only age in years at the time of last transplant and the number of surgical complications after the last transplant had a significant influence on patient survival during follow-up (*P* = 0.016, hazard ratio 1.069, 95% confidence interval 1.012–1.128 and *P* = 0.029, hazard ratio 2.275, 95% confidence interval 1.088–4.757, resp.) (Tables 2 and 3). The distribution of age in years was not significantly different for different numbers of surgical complications after the last transplant (*P* = 0.446, ANOVA, pairwise Kruskal-Wallis test). Risk-adjusted analysis revealed that only age at the time of last transplant had an independent significant influence on patient survival (*P* = 0.016, hazard ratio 1.068, 95% confidence interval 1.012–1.128, multivariate Cox regression).

**Figure 1:**
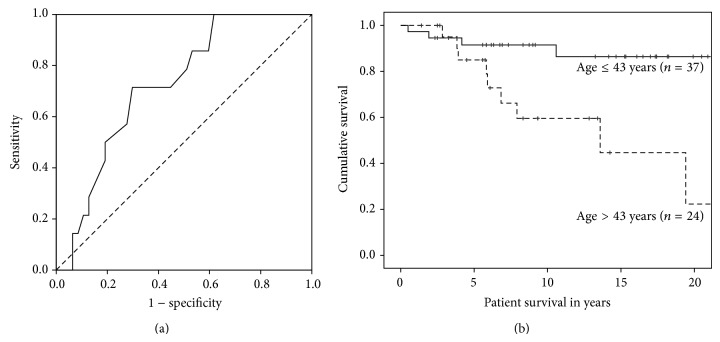
(a) The ROC-curve for age in years is shown for the prediction of mortality during follow-up. The area under the curve (AUROC) indicates potential prognostic value (AUROC = 0.723; 95% CI: 0.588–0.857). Multivariate Cox regression revealed age as a significant independent risk factor for mortality during follow-up. The cut-off value for age in years at last transplant chosen with the best Youden index for the prediction of mortality was 43 years. (b) Survival of patients who are ≤ 43 years old is significantly superior as compared to those who are older than 43 years at the time of their last kidney transplant (*P* = 0.005; Kaplan-Meier survival analysis, log rank test).

ROC-curve analysis of age in years at the last transplant for the prediction of mortality during follow-up revealed an area under the curve (AUROC) with potential prognostic value (AUROC = 0.723; 95% CI: 0.588–0.857). Based on these ROC-curve results the cut-off value for age in years at last transplant chosen with the best Youden index for the prediction of mortality after multiple kidney transplants was 43 years (sensitivity 71.4%, specificity 70.2%, and overall correctness of prediction 70.8%) (Figure 1(a)). Age above 43 years had a statistically significant influence on patient survival in years as demonstrated in Figure 1(b) (*P* = 0.005; Kaplan-Meier analysis with log rank test). The distribution of recipient blood groups was significantly different in patients older than 43 years as compared to younger patients at the time of transplant while the distributions of all other variables were not significantly different.

### 3.2. Graft Survival

**Figure 2:**
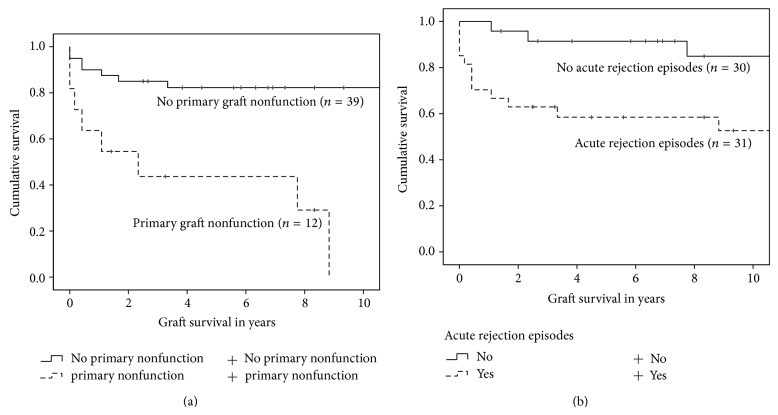
The influences of a primary graft nonfunction (a) and acute allograft rejection episodes during follow-up (b) on graft survival censored for death with functioning graft are shown (Kaplan-Meier survival analysis). The log rank test reveals that the differences in graft survival are statistically significant (*P* < 0.001 and *P* = 0.002, resp.).

Actuarial graft survival was 86.8% after one year, 80.0% after three years, and 78.1% after 5 years, respectively. Univariate Cox regression showed that only the number of all HLA mismatches, HLA mismatches in groups (0–2 versus 3–6), cyclosporine versus tacrolimus based initial immunosuppression, the number of surgical complications, reoperation due to a complication (yes/no), primary graft nonfunction (yes/no), and acute rejection episodes (yes/no) after the last transplant had a significant influence on graft survival (Table 4). Risk-adjusted analysis revealed that only primary graft nonfunction and acute rejection had an independent significant influence on graft survival (*P* = 0.001, hazard ratio 5.890, 95% confidence interval 2.059–16.853 and *P* = 0.017, hazard ratio 3.944, 95% confidence interval 1.278–12.174, resp.). Primary graft nonfunction and acute allograft rejection episodes did not influence each other significantly (two-sided asymptotic significance *P* = 0.221, Pearson's *χ* ^2^ test). As expected, primary graft nonfunction and acute allograft rejection episodes during follow-up had significant influences on time to graft loss censored for death with functioning graft (*P* < 0.001 and *P* = 0.002, resp.; log rank test) (Figures 2(a) and 2(b)) Table 5.

**Figure 3:**
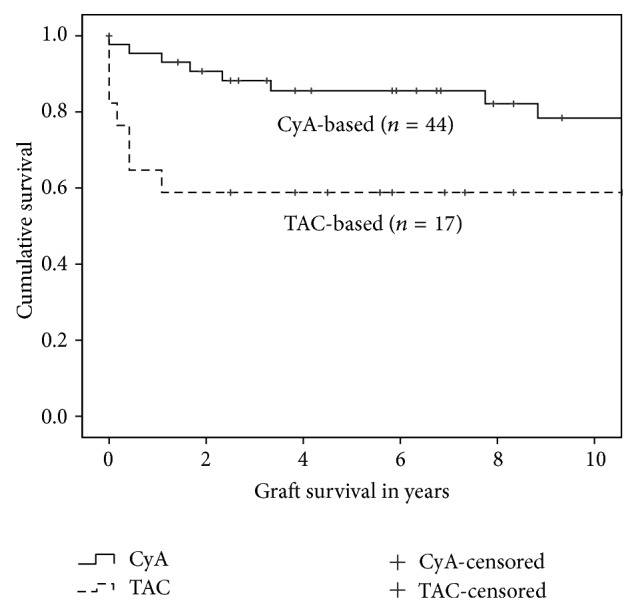
The significant influence of a primary immunosuppressive protocol based on cyclosporine versus a primary immunosuppressive protocol based on tacrolimus on graft survival is shown (*P* = 0.016; Kaplan-Meier survival analysis with log rank test).

Time to graft loss was significantly influenced by cyclosporine versus tacrolimus based immunosuppression therapy with significantly shortened time to graft loss for patients treated with tacrolimus (*P* = 0.016, log rank test) (Figure 3). This influence of cyclosporine versus tacrolimus based initial immunosuppression could not be confirmed as an independent risk factor for graft survival in risk-adjusted multivariate Cox regression analysis. The distribution of the numbers of performed kidney transplants in each patient (*n* = 3 73.5% versus *n* = 4 88.9% versus *n* = 5 0%), performed perioperative plasmapheresis treatments (56.0% versus 44.0%), the number of previous operations (mean 1.77, range 1–3 versus mean 2.06, range 0–4), and the occurrence of acute allograft rejection episodes (58.1% versus 41.9%, 65% antibody mediated and 35% T-cell mediated) were all significantly different in patients treated with cyclosporine as compared to those with tacrolimus (*P* = 0.011, *P* = 0.019, and *P* = 0.003 and 0.013, resp.; two-sided asymptotic significance, Pearson's *χ* ^2^ test) while the distributions of all other variables were not significantly different.

### 3.3. Surgical Complications

The overall rate of postoperative surgical complications was 44%. More specifically 27 of the renal transplant recipients (44%) developed 36 surgical complications and 13 of them (21.3%) had to be reoperated because of a surgical complication. Based on the Clavien classification system of postoperative complications, 10 were grade 1, eight grade 2, four grade 3a, and fourteen grade 3b. No recipient suffered a life-threatening complication and no renal graft was lost as a result of a surgical complication. The most common complication (30%) was postoperative wound infection which was observed in 11 patients, followed by nine cases of lymphocele (25%) and 6 cases of urinary leckage (17%). Out of the 13 recipients, who had to be reoperated because of a surgical complication, 4 patients underwent renewal of the ureteroneocystostomy, 3 patients a fenestration of lymphocele, and 3 patients revision of the wound. Early surgical revision was required in only one patient because of an intestinal perforation.

## 4. Discussion

Renal transplantation is the best treatment for patients suffering from end-stage renal disease, but with an estimated half-life of about nine years for primary deceased donor kidney grafts; it is obvious that many recipients will face graft loss, will return to dialysis treatment, and eventually will be considered for a retransplantation. As failure of renal graft constitutes a risk factor associated with higher recipient mortality and morbidity in patients returning to chronic dialysis treatment, a retransplantation seems to be the best treatment for this group of patients. On the other hand, living in a period of shortage of organs it has to be questioned whether it is acceptable to offer to all of these patients a retransplantation, taking into consideration the high-risk characteristics of this population. The aim of this study was to assess the patient and graft survival after second or subsequent renal transplantation and to identify risk factors associated with inferior outcomes and loss of renal graft.

This single center study presented a patient survival of 98.3% after one year, 94.8% after three years, and 88.2% after five years for the entire cohort of the 61 patients transplanted between January 1990 and December 2010. As the statistical analysis showed that the number of previous renal transplantations had no effect on patient survival and because of the small number of patients after fourth (*n* = 9) and fifth (*n* = 3) renal transplantation, patient survival for the subgroups of patients was not analyzed. The patient survival rates mentioned above correlate with the results presented by almost all of the actual studies [16, 19–21] and they do not differ significantly from the overall patient survival rates after renal transplantation as presented in our previous study [22]. Regarding the factors that could influence the survival of the renal transplant recipients, the univariate analysis of our data showed that only age in years at the time of the last transplantation and the number of surgical complications after the last transplant had a significant influence on patient survival during follow-up. Moreover, the risk-adjusted analysis revealed that only age of renal transplant recipients had an independent significant influence on patient survival. Using the ROC-curve analysis for age in years, it was shown that renal transplant recipients younger than 43 years of age at the last transplantation had a significantly superior survival as compared to those who were older than 43 years at the time of their last kidney transplantation. Our study is the first one that presents a cut value for age in years as an independent risk factor for mortality after follow-up. According to our opinion, our results and especially the influence of the age on patient survival could be attributed to the comorbidities of the recipients, especially hypertension, atherosclerosis, and cardiovascular disease, developed because of the prolonged time spent on dialysis treatment [23–25].

Regarding the graft survival, our study presented a survival of 86.8% after one year, 80.0% after three years, and 78.1% after five years, respectively, for the entire cohort of the sixty-one renal transplant recipients. Again, the number of previous renal transplantations had no effect on graft survival and the graft survival for the subgroups of patients was not analyzed. The univariate analysis of these data revealed, in contrast to the patient survival, that many factors had a significant influence on the graft survival of our study population. It was shown that immunological aspects such as the number of the HLA mismatches and the occurrence of acute rejections episodes, the type of immunosuppression (cyclosporine versus tacrolimus), and primary graft nonfunction but also the occurrence of surgical complications with need of reoperation were associated with poor graft survival after second or subsequent renal transplantation. On the other hand, the risk-adjusted analysis revealed that only primary graft nonfunction and acute rejection episodes had an independent significant influence on graft survival. These results reflect the major difficulties faced in patients receiving multiple renal transplantations, which is in line with other studies [19–21, 26], consisting of the high sensitization of the patients and the surgical difficulties due to the previous retransplantation and previous transplant nephrectomies.

In conclusion, a third or subsequent renal transplantation constitutes nowadays a valid therapeutic option with acceptable patient and graft survival rates but always taking into consideration the major risk factors shown in our study such as the age of the recipients, the immunological components, and the occurrence of postoperative surgical complications. Therefore, every single patient should be evaluated individually and loss of previous renal grafts should not be an exclusion criterion for patients on the waiting list for retransplantation.

## Figures and Tables

**Table 1:** The distributions of variables in the investigated cohort are shown.

Distribution of recipient variables	*n*	Mean (median)	SD	Range
Number and percentage of female patients	29 (47.5%)	n.a.	n.a.	n.a.
Blood groups				
0	15 (24.6%)	n.a.	n.a.	n.a.
A	35 (57.4%)			
B	2 (3.3%)			
AB	9 (14.8%)			
Age at last transplant in years	n.a.	39.3 (39)	10.8	20–63
Number of kidney transplants/patient	n.a.	3.3 (3)	0.5	3–5
PRA max. in %	n.a.	72.8 (81.0)	29.0	0–100
PRA max. in groups				
0–30%	7 (11.5%)			
>30–70%	16 (26.2%)	n.a.	n.a.	n.a.
>70%	38 (62.3%)			
PRA at transplant in %	n.a.	43.7 (40)	38.4	0–100
PRA at transplant in groups				
0–30%	23 (37.7%)			
>30–70%	17 (27.9%)	n.a.	n.a.	n.a.
>70%	21 (34.4%)			
Number of HLA-DR mismatches				
0	33 (54.1%)			
1	21 (34.4%)	n.a.	n.a.	n.a.
2	7 (11.5%)			
Number of all HLA mismatches	n.a.	1.8 (2)	1.5	0–5
Number of all HLA mismatches in groups				
0–2	39 (63.9%)	n.a.	n.a.	n.a.
3–6	22 (36.1%)			
Plasmapheresis	25 (41.0%)	n.a.	n.a.	n.a.
Induction therapy	46 (75.4%)	n.a.	n.a.	n.a.
Type of induction therapy:				
Basiliximab (yes/no)	15 (24.1%)	n.a.	n.a.	n.a.
Thymoglobulin (yes/no)	31 (50.8%)			
Cyclosporine based protocol	44 (72.1%)	n.a.	n.a.	n.a.
Tacrolimus based protocol	17 (27.9%)	n.a.	n.a.	n.a.
Mycophenolate mofetil therapy	56 (91.8%)	n.a.	n.a.	n.a.
Azathioprine therapy	5 (8.2%)	n.a.	n.a.	n.a.
Living donation	2 (3.3%)	n.a.	n.a.	n.a.
Postmortem donation after brain death	59 (96.7%)	n.a.	n.a.	n.a.
Simultaneous nephrectomy of previous graft	14 (23.0%)	n.a.	n.a.	n.a.
Number of previous operations	n.a.	1.9 (2)	0.7	0–4
Operating time in min	n.a.	163.8 (160)	59.6	70–390
Cold ischemic time in min	n.a.	1140 (1175)	384	105–2104
Number of surgical complications	n.a.	0.6 (0)	0.8	0–3
Reoperations due to complications	13 (21.3%)	n.a.	n.a.	n.a.
Primary graft non-function	12 (19.7%)	n.a.	n.a.	n.a.
Acute graft rejection episodes	31 (50.8%)	n.a.	n.a.	n.a.
Chronic graft rejection	4 (6.6%)	n.a.	n.a.	n.a.

SD = standard deviation; n.a. = not applicable.

**Table 2:** The univariate influences of the investigated variables on patient survival are shown.

Recipient variables	*P* value	Hazard ratio	95% confidence interval
Recipient sex	0.191	n.a.	n.a.
Recipient blood group	0.547	n.a.	n.a.
Age in years at last transplantation	0.016	1.068	1.012–1.128
Number of kidney transplants	0.583	n.a.	n.a.
PRA max. in %	0.231	n.a.	n.a.
PRA max. in groups (0–30%, >30–70%, and >70%)	0.386	n.a.	n.a.
PRA at last transplant in %	0.607	n.a.	n.a.
PRA at last transplant in groups (0–30%, >30–70%, and >70%)	0.578	n.a.	n.a.
Number of HLA-DR mismatches	0.918	n.a.	n.a.
Number of all HLA mismatches	0.656	n.a.	n.a.
Number of all HLA mismatches in groups (0–2, 3–6)	0.348	n.a.	n.a.
Plasmapheresis (yes/no)	0.719	n.a.	n.a.
Induction therapy (yes/no)	0.321	n.a.	n.a.
Type of induction therapy	0.180	n.a.	n.a.
Cyclosporine versus tacrolimus based therapy	0.066	n.a.	n.a.
Mycophenolate mofetil versus azathioprine therapy	0.430	n.a.	n.a.
Living donor versus deceased donor	0.482	n.a.	n.a.
Simultaneous nephrectomy of previous graft (yes/no)	0.532	n.a.	n.a.
Number of previous operations at transplant site	0.265	n.a.	n.a.
Operating time in minutes	0.359	n.a.	n.a.
Cold ischemic time in minutes	0.632	n.a.	n.a.
Surgical complications (yes/no)	0.294	n.a.	n.a.
Number of surgical complications	0.029	2.275	1.088–4.757
Reoperation due to a complication (yes/no)	0.054	n.a.	n.a.
Primary graft non-function (yes/no)	0.998	n.a.	n.a.
Acute graft rejection episodes (yes/no)	0.267	n.a.	n.a.
Chronic graft rejection (yes/no)	0.487	n.a.	n.a.
Graft loss (yes/no)	0.563	n.a.	n.a.

Univariate Cox regression analysis, n.a. = not applicable.

**Table 3:** Those variables with significant influences on patient survival in univariate Cox regression analysis and their independent influences on patient survival in multivariate Cox regression analysis are shown.

Recipient variables	*P* value	Hazard ratio	95% confidence interval
Age in years	0.016	1.068	1.012–1.128
Number of surgical complications	0.077	n.a.	n.a.

n.a. = not applicable.

**Table 4:** The univariate influences of the investigated variables on graft survival are shown (univariate Cox regression analysis).

Recipient variables	*P* value	Hazard ratio	95% confidence interval
Recipient sex	0.604	n.a.	n.a.
Recipient blood group	0.562	n.a.	n.a.
Age in years at last transplantation	0.433	n.a.	n.a.
Number of kidney transplants	0.653	n.a.	n.a.
PRA max. in %	0.460	n.a.	n.a.
PRA max. in groups (0–30%, >30–70%, and >70%)	0.480	n.a.	n.a.
PRA at last transplant in %	0.412	n.a.	n.a.
PRA at last transplant in groups (0–30%, >30–70%, and >70%)	0.427	n.a.	n.a.
Number of HLA-DR mismatches	0.441	n.a.	n.a.
Number of all HLA mismatches	0.004	1.657	1.180–2.326
Number of all HLA mismatches in groups (0–2, 3–6)	0.002	4.440	1.691–11.655
Plasmapheresis (yes/no)	0.410	n.a.	n.a.
Induction therapy (yes/no)	0.334	n.a.	n.a.
Type of induction therapy	0.261	n.a.	n.a.
Cyclosporine versus tacrolimus based therapy	0.028	3.176	1.131–8.916
Mycophenolate mofetil versus azathioprine therapy	0.839	n.a.	n.a.
Living donor versus deceased donor	0.374	n.a.	n.a.
Simultaneous nephrectomy of previous graft (yes/no)	0.535	n.a.	n.a.
Number of previous operations at transplant site	0.463	n.a.	n.a.
Operating time in minutes	0.958	n.a.	n.a.
Cold ischemic time in minutes	0.914	n.a.	n.a.
Surgical complications (yes/no)	0.059	n.a.	n.a.
Number of surgical complications	0.003	2.225	1.318–3.758
Reoperation due to a complication (yes/no)	0.026	3.013	1.144–7.939
Primary graft nonfunction (yes/no)	<0.001	6.384	2.290–17.797
Acute graft rejection episodes (yes/no)	0.005	4.872	1.602–14.816
Chronic graft rejection (yes/no)	0.888	n.a.	n.a.

**Table 5:** The univariate influences of the investigated variables on primary nonfunction of the graft are shown (univariate binary logistic regression analysis).

Recipient variables	*P* value	Odds ratio	95% confidence interval
Recipient sex	0.849	n.a.	n.a.
Recipient blood group	0.167	n.a.	n.a.
Age in years at last transplantation	0.374	n.a.	n.a.
Number of kidney transplants	0.526	n.a.	n.a.
PRA max. in %	0.942	n.a.	n.a.
PRA max. in groups (0–30%, >30–70%, and >70%)	0.609	n.a.	n.a.
PRA at last transplant in %	0.706	n.a.	n.a.
PRA at last transplant in groups (0–30%, >30–70%, and >70%)	0.323	n.a.	n.a.
Number of HLA-DR mismatches	0.024	2.867	1.150–7.148
Number of all HLA mismatches	0.016	1.827	1.120–2.982
Number of all HLA mismatches in groups (0–2, 3–6)	0.004	8.308	1.944–35.502
Plasmapheresis (yes/no)	0.479	n.a.	n.a.
Induction therapy (yes/no)	0.998	n.a.	n.a.
Type of induction therapy	0.010	4.316	1.422–13.100
Cyclosporine versus tacrolimus based therapy	0.234	n.a.	n.a.
Mycophenolate mofetil versus azathioprine therapy	0.248	n.a.	n.a.
Living donor deceased donor	0.477	n.a.	n.a.
Simultaneous nephrectomy of previous graft (yes/no)	0.851	n.a.	n.a.
Number of previous operations at transplant site	0.334	n.a.	n.a.
Operating time in minutes	0.796	n.a.	n.a.
Cold ischemic time in minutes	0.763	n.a.	n.a.
Surgical complications (yes/no)	0.003	24.933	2.941–210.938
Number of surgical complications	0.001	7.223	2.206–23.648
Reoperation due to a complication (yes/no)	0.257	n.a.	n.a.
Acute graft rejection episodes (yes/no)	0.221	n.a.	n.a.
Chronic graft rejection (yes/no)	0.782	n.a.	n.a.

## References

[1] Hricik D. E., Halbert R. J., Barr M. L. (2001). Life satisfaction in renal transplant recipients: preliminary results from the transplant learning center. *American Journal of Kidney Diseases*.

[2] Wolfe R. A., Ashby V. B., Milford E. L. (1999). Comparison of mortality in all patients on dialysis, patients on dialysis awaiting transplantation, and recipients of a first cadaveric transplant. *The New England Journal of Medicine*.

[3] Kaplan B., Meier-Kriesche H.-U. (2002). Death after graft loss: an important late study endpoint in kidney transplantation. *The American Journal of Transplantation*.

[4] Oniscu G. C., Brown H., Forsythe J. L. R. (2005). Impact of cadaveric renal transplantation on survival in patients listed for transplantation. *Journal of the American Society of Nephrology*.

[5] Costa-Requena G., Aixendri M. C. C., Urrutia A. R., Micas D. S. (2014). Health related quality of life and kidney transplantation: a comparison with population values at 6 months post-transplant. *Medicina Clínica*.

[6] Lamb K. E., Lodhi S., Meier-Kriesche H. U. (2011). Long-term renal allograft survival in the United States: a critical reappraisal. *The American Journal of Transplantation*.

[7] Meier-Kriesche H.-U., Schold J. D., Kaplan B. (2004). Long-term renal allograft survival: have we made significant progress or is it time to rethink our analytic and therapeutic strategies?. *American Journal of Transplantation*.

[8] Meier-Kriesche H.-U., Schold J. D., Srinivas T. R., Kaplan B. (2004). Lack of improvement in renal allograft survival despite a marked decrease in acute rejection rates over the most recent era. *American Journal of Transplantation*.

[9] Miles C. D., Schaubel D. E., Jia X., Ojo A. O., Port F. K., Rao P. S. (2007). Mortality experience in recipients undergoing repeat transplantation with expanded criteria donor and non-ECD deceased-donor kidneys. *The American Journal of Transplantation*.

[10] Rao P. S., Schaubel D. E., Wei G., Fenton S. S. A. (2006). Evaluating the survival benefit of kidney retransplantation. *Transplantation*.

[11] Ojo A. O., Wolfe R. A., Agoda L. Y. (1998). Prognosis after primary renal, transplant failure and the beneficial effects of repeat transplantation: multivariate analyses from the United States renal data system. *Transplantation*.

[12] Loupy A., Anglicheau D., Timsit M. O. (2007). Impact of surgical procedures and complications on outcomes of third and subsequent kidney transplants. *Transplantation*.

[13] Hagan C., Hickey D. P., Little D. M. (2003). A single-center study of the technical aspects and outcome of third and subsequent renal transplants. *Transplantation*.

[14] Mazzucchi E., Danilovic A., Antonopoulos I. M. (2006). Surgical aspects of third and subsequent renal transplants performed by the extraperitoneal access. *Transplantation*.

[15] Blanco M., Medina J., Gonzalez E. (2009). Third kidney transplantation: a permanent medical-surgical challenge. *Transplantation Proceedings*.

[16] Horovitz D., Caumartin Y., Warren J. (2009). Outcome of third renal allograft retransplants versus primary transplants from paired donors. *Transplantation*.

[17] Ahmed K., Ahmad N., Khan M. S. (2008). Influence of number of retransplants on renal graft outcome. *Transplantation Proceedings*.

[18] Pour-Reza-Gholi F., Nafar M., Saeedinia A. (2005). Kidney retransplantation in comparison with first kidney transplantation. *Transplantation Proceedings*.

[19] Izquierdo L., Peri L., Piqueras M. (2010). Third and fourth kidney transplant: still a reasonable option. *Transplantation Proceedings*.

[20] Loupya A., Anglicheau D., Suberbielle C. (2007). Long-term outcome of third kidney transplants. *Nephrology Dialysis Transplantation*.

[21] Kienzl-Wagner K., Mark W., Maglione M. (2011). Single-center experience with third and fourth kidney transplants. *Transplant International*.

[22] Kousoulas L., Emmanouilidis N., Gwinner W., Klempnauer J., Lehner F. (2013). High-urgency renal transplantation: indications and long-term outcomes. *Journal of Transplantation*.

[23] Kutyrina I. M., Rudenko T. E., Savelyeva S. A., Shvetsov M. Y., Vasilyeva M. P. (2013). Risk factors for cardiovascular system damage in chronic kidney disease. *Terapevticheskii Arkhiv*.

[24] Chantrel F., de Cornelissen F., Deloumeaux J., Lange C., Lassalle M., Registre REIN (2013). Survival and mortality in ESRD patients. *Néphrologie & Thérapeutique*.

[25] Kahn M. R., Robbins M. J., Kim M. C., Fuster V. (2013). Management of cardiovascular disease in patients with kidney disease. *Nature Reviews Cardiology*.

[26] Halawa A. (2012). The third and fourth renal transplant; technically challenging, but still a valid option. *Annals of Transplantation*.

